# The safety and effectiveness of a novel annular keratopigmentation technique; a cross-sectional survey of patients

**DOI:** 10.1186/s12886-023-02911-7

**Published:** 2023-06-27

**Authors:** Munirah Alafaleq, Robert van Haselen, Francis Ferrari

**Affiliations:** 1 Ophthalmology Department, AP-HP, University Hospital Necker-Enfants Malades, University of Paris, Paris, France; 2 Ophthalmology department, Imam Abdulrahman Bin Faisal University, King Fahd Hospital of the University, P. O. Box 1982, Dammam, 31441 Saudi Arabia; 3 International Institute for Integrated Medicine, Richmond, UK; 4 Clinique Espace Nouvelle Vision, 6 Rue de la Grande Chaumière, Paris, 75006 France

**Keywords:** Femtosecond assisted aesthetic annular keratopigmentation, Procedural safety, Corneal tattoo

## Abstract

**Purpose:**

We investigated safety aspects and other patient experiences related to a novel Femtosecond Laser Assisted Annular Keratopigmentation technique (FLAAK).

**Setting:**

Espace Nouvelle Vision Clinic in Paris.

**Methods:**

Monocentric, post-operative, cross-sectional survey of patients who returned to the clinic for a color correction after the FLAAK procedure. Whilst waiting for their color retouch, consenting patients completed a questionnaire about their experiences following the FLAAK procedure. Aspects related to side-effects or discomfort as well as patient satisfaction were assessed.

**Results:**

The questionnaire was completed by 42 of 51 patients returning to the clinic for a color retouch (27 females, 15 males; mean age 37.6 years). Pain was experienced by 34 (81%) patients, dry eyes by 32 (76%) patients, glare by 23 (56%) patients, red eyes by 28 (67%) patients, and tingling by 30 (71%) patients; no patient experienced visual halos. All experienced post-operative symptoms were of a transient nature. Symptoms like pain, tingling, glare and red eyes disappear in less than 48 h after surgery in approximately 50% of the cases, and ocular dryness in 22% of cases., The median duration of these symptoms in patients for whom the symptoms were still present after 48 h, is 7 days. Patient satisfaction with the aesthetical result (scale ranging from 0 to 10) was on average 8,1 (SD 1,6).

**Conclusion:**

The FLAAK procedure performed for purely aesthetic purposes appears to be safe and is associated with high patient satisfaction.

## Introduction

An increasing number of patients are seeking permanent eye color change procedures. Three techniques are currently available: cosmetic iris implants, laser iris depigmentation and Femtosecond Laser Assisted Annular Keratopigmentation (FLAAK). Cosmetic implants are now known for their serious complications [1]. Laser iris depigmentation seems to be a safer technique, but complications were recently reported [2, 3, 4, 5]. FLAAK was first described by Aliò et al. for pathological eyes in 2011 [6], and by Ferrari for cosmetic reasons in 2015 [7].

Corneal tattooing, or keratopigmentation, was first mentioned by Galen in the 1st century [8]. Such techniques were principally used for therapeutic purposes, in patients with iris defects [6, 9, 10], Corneal scars [11, 12], leukocoria [13], strabismic diplopia [14], and Urrets-Zavalia syndrome [15]. Further improvements in surgical techniques as well as the dyes used, have led to an increased interest in corneal tattooing for aesthetic purposes in recent years [16].

Little is known however about the safety and effectiveness in healthy human subjects.

We previously reported on the safety and effectiveness of a novel esthetic keratopigmentation method in a clinical case report [17]. This technique consists first of creating an intrastromal annular tunnel with a femtosecond laser, and then the dissection is made with a special round spatula followed up by the insertion of a micronized mineral pigment (Biotic Phocéa, Marseille, France).

In order to enable subjects to make informed decisions with regard to this procedure, a research program to further investigate the safety and effectiveness is in development. We report on the results of a survey of patients who were treated by this novel technique.

## Materials and methods

Cross-sectional survey of patients returning for a color retouch following annular keratopigmentation using the FLAAK procedure. Patients attending the Espace Nouvelle Vision Clinic in Paris from 17/04/2019 to 04/11 2020 were eligible.

The questionnaire contained questions related to demographics, experiences with prior aesthetic eye surgery (if applicable), reason(s) for seeking an eye color correction, experienced symptoms/discomfort after the procedure, and various aspects related to satisfaction with treatment outcome. Overall patient satisfaction with the results of the procedure was rated on a scale from zero to 10. Additionally, the subjects were asked to indicate if their general wellbeing had improved since the procedure on a 5-point scale ranging from 0 (no amelioration) to 4 (greatly ameliorated).

Paper questionnaires were completed whilst, or the day before, patients were attending the clinic in preparation of their retouch procedure. The questionnaires were checked and entered by the clinic assistant. Logical checks of the data took place and any queries were resolved on the basis of further discussion during data-management. After database lock, the data were analyzed descriptively. Continuous data are presented as the mean ± standard deviation and/or the median as appropriate. Categorical data are presented as numbers and percentages of the relevant total. Statistical analysis was performed using Microsoft Excel for Mac (version 14.7.7).

### Patient and public involvement

Patients were not involved in the design, or conduct, or reporting, or dissemination plans of our research.

## Results

Forty-two of 51 eligible patients who attended the clinic during the study period, completed the questionnaire. Of the remaining 9 patients, one did not consent (no specific reason given) and the remaining 8 patients were not offered the questionnaire due to lack of time and/or other logistical reasons. Therefore, the response rate of those who were offered the questionnaire was 98%.

The questionnaires were completed on average 13.4 months (SD 7.8, range 5–38) after the initial FLAAK procedure.

Demographic and baseline characteristics of the sample are presented in Table 1.

**Table 1:** Demographic, clinical and other relevant characteristics

Characteristics	Value*
N respondents	42
Age in years (SD)	37.6 (8.6)
Sex (female / male)	64.3 / 35.7
Country of Residence (France / other EU countries / non-EU countries)	42.8 /28.6 / 28.6
Educational attainment (up to high school / higher education / student)	30.9 / 66.6 / 2.5
Employment status (employed / student / other)	80.9 / 4.8 / 14.3
Aesthetic surgery prior to Neoris procedure (yes / no)	26.8 / 73.2
Used other methods for changing eye colour (yes / no)	28.5 / 71.5
Colored lenses used prior to Neoris procedure (yes / no)	54.7 / 43.3
Reason(s) for seeking Neoris procedure (feeling better in skin / being more attractive / boosting self confidence / other)	35.0 / 27.7 / 21.1 / 16.2

* Values are percentages, unless otherwise mentioned

A majority of subjects (64%) were female, and had completed higher professional education (67%). A significant minority had previous other types of aesthetic surgery (27%). A majority of patients (55%) had previously used coloured lenses for a median period of 10 years (range 1–20) prior to seeking treatment. 29% of patients had previously used/tried other methods for changing eye colour, most commonly this involved laser depigmentation or color implants. The most common reasons for seeking a change of eye colour were related to increasing confidence about one’s appearance.

**Fig. 1:**
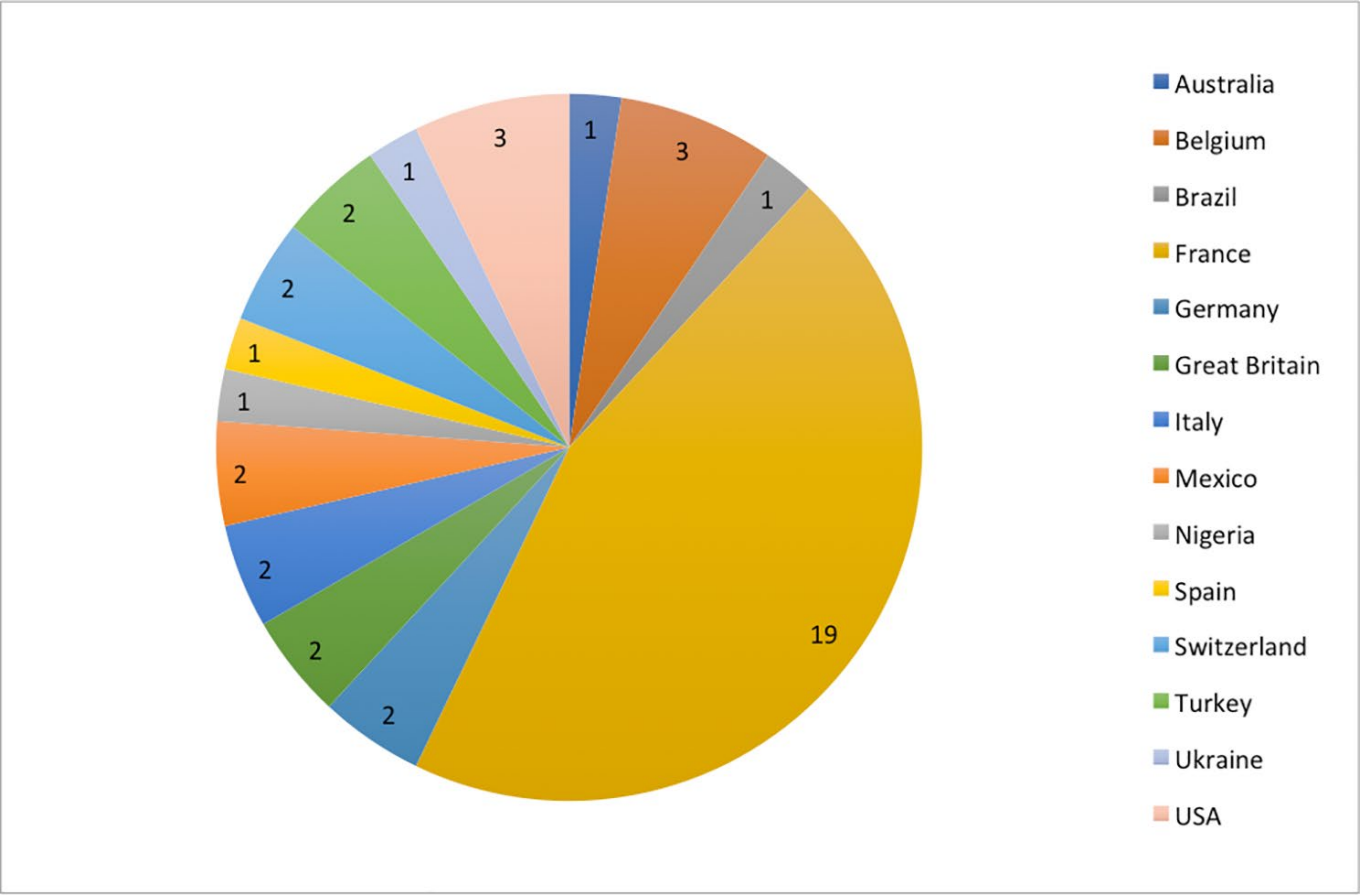
The broad range of nationalities seeking the procedure

The broad range of nationalities seeking the procedure is illustrated in Fig. 1.

**Fig. 2:**
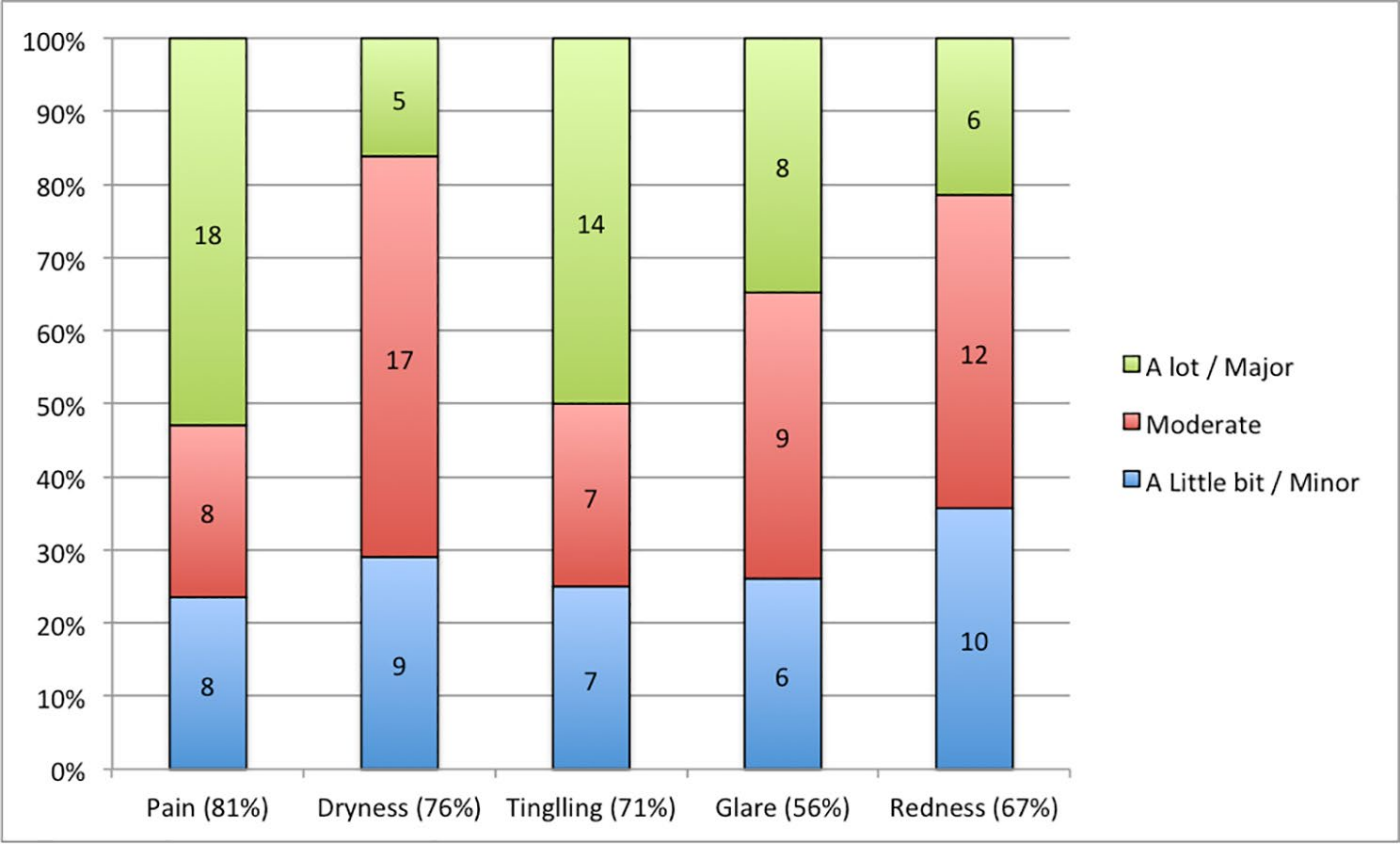
Safety related data

Safety related data are depicted in Fig. 2.

Pain, dryness, tingling, redness, and glare were the most common symptoms experienced peri- and post-operatively by patients (Fig. 2).

In 25 of 34 patients (81%) who experienced pain, this occurred on the day of and/or after the procedure. The median duration of pain in the 9 patients that experienced pain longer, was 6.5 days, including two outlier patients that reported experiencing pain for 120 and 270 days respectively.

In 7 of 32 patients (22%) who experienced eye dryness, this occurred on the day of and/or after the procedure. The median duration of eye dryness for the patients who experienced dryness longer, was 7 days for the patients who started the dryness the day of the intervention, and 18 days for the patients who started the dryness the day after the intervention.

In 17 of 30 patients (57%) who experienced tingling, this occurred only on the day of and/or after the procedure. The median duration of tingling for the patients who experienced tingling longer, was 6 days. In one patient the tingling started the day after the surgery and lasted for 60 days.

In 11 of 23 patients (48%) who experienced glare, this occurred only on the day of and/or after the procedure. The median duration of glare in patients who experienced glare longer, was 7 days.

In 14 of 28 patients (50%) who experienced red eyes, this occurred only on the day of and/or after the procedure. The median duration of red eyes in patients who experienced red eyes longer, was 7 days.

**Fig. 3:**
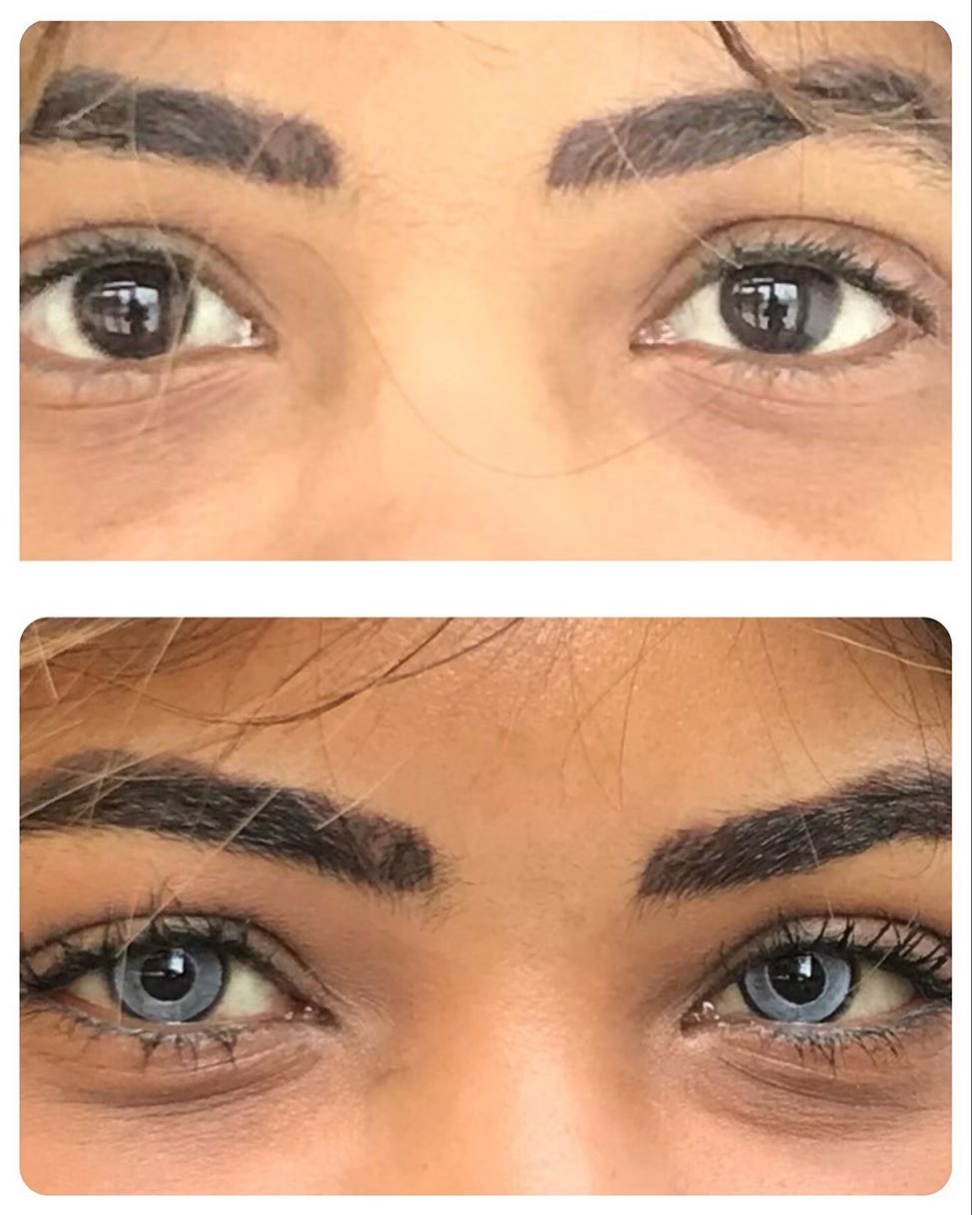
An example of the post- versus pre-operative appearance of the eyes of one of the patients (patient consented to publication)

Patient satisfaction with the aesthetical result (scale ranging from 0 to 10) was on average 8,1 (SD 1.6). An example of the post- versus pre-operative appearance of the eyes of one of the patients is given in Fig. 3 (patient consented to publication).

Patients also reported an improvement in wellbeing after the procedure of on average 2.8 (SD 1.3) on a scale ranging from zero to four. Only two patients reported ‘no amelioration’ in wellbeing. Under the -conservative- assumption that the 4 patients with missing values for this variable all experienced ‘no amelioration’, 36 (85%) of the patients reported experiencing an improvement in wellbeing since the procedure.

## Discussion

In this survey we evaluated the safety and satisfaction of patients who returned to the clinic for a color retouch after FLAAK. We found that the majority of patients experienced some symptoms, principally on the day of, and after, the surgery. Exceptionally, patients reported experiencing pain and tingling for several months after surgery. All experienced symptoms resolved completely, and no serious adverse events were observed or reported. Overall, satisfaction with the results of the procedure was high.

This was the first survey of patients from various countries who had undergone a novel FLAAK corneal tattooing technique. A strength of this study is the very high response rate (98%), effectively ruling out selection bias in the study sample.

A weakness of our study is that the sampled retouch population may not be fully representative of the approximately 47% of patients that do not return for a retouch procedure. Also, the size of the study sample was relatively small. Furthermore, some recall bias cannot be ruled out.

Francesco D’Oria et al. [16] assessed patient satisfaction after purely cosmetic keratopigmentation in 40 patients and reported a satisfactory outcome in 93% of cases. The high percentage of satisfied patients was similar to the findings in our study.

Permanent change of eye color for purely cosmetic reason can be performed by three methods: cosmetic iris implants, iris laser depigmentation and by using a femtosecond laser. Iris implants have been associated with serious complications including, severe vision threatening, glaucoma, corneal endothelial cell decompensation and uveitis-glaucoma-hyphema syndrome [18–22].

Even though the majority of patients in our study (65%) felt that color iris implants were more aesthetic, they still chose FLAAK procedure because they considered it safer.

Laser iris depigmentation has been used clinically for aesthetic purposes without receiving official approval or licensing. The procedure uses a device mounted onto a slit-lamp biomicroscope that produces a frequency-doubled 532-nm wavelength neodymium: yttrium–aluminum–garnet (Nd:YAG) laser with different spot diameters which are focused on the anterior iris stroma. Although this technique seems to be safer than the cosmetic implant, we reported a case of iris perforation after excessive laser iris depigmentation. [2] Additionally, a major concern with regard to this technique is its lack of effectiveness: on dark pigmented eyes, even after more than 10 laser sessions, the result is generally unnoticeable, except when the subject’s eyes are in direct sunlight. As a result, we are performing a lot of keratopigmentations on patients who are dissatisfied after laser iris depigmentation.

The tolerance of the pigment used in FLAAK is good [23]. In some cases changes in the color over time have been reported, that were most likely due to some iron-based components in the pigment. Optimized dyes, which are devoid of molecules potentially responsible for fading or changes in color, are available on the market since 2022 (Neoris, Paris, France).

FLAAK seems to be the safest and most effective procedure. To date (June 2022) we conducted more than 800 procedures without any serious complications. In the great majority of our patients, the perceived benefits of increasing aesthetic appearance and higher self-confidence outweighed any experienced side-effects or discomfort. Compared to the two other techniques, the procedure appears to have a positive risk-benefit ratio in the majority of patients.

In this study we focused only on patients undergoing a color retouch, therefore patients not undergoing a color retouch were not included. A prospective observational study that includes both retouch and non-retouch patients with an adequate sample size should be considered as a next step of the research program.

The current study confirms that the majority of patients prefer FLAAK surgery because they consider it safer and more effective. Hence the need to further and systematically review safety aspects. This is an important innovation in the field of cornea surgery, which also has potential in the treatment of patients with ocular pathologies [6, 15, 24].

## Data Availability

The datasets used and/or analysed during the current study, are available from the corresponding author on reasonable request.

## References

[1] George MK, Tsai JC, Loewen NA (2011). Bilateral irreversible severe vision loss from cosmetic iris implants. Am J Ophthalmol.

[2] Ferrari F (2021). Case report of an iris perforation after YAG laser iris depigmentation for aesthetic reasons]. J Fr Ophtalmol.

[3] Ning Brigid, Baboolal S, Gizzi C, Nolan W. A Case of Secondary Pigment Dispersion Following Laser to Cosmetically Lighten the Irises. J Glaucoma. 2022 Feb 1;31(2):133–135. 10.1097/IJG.0000000000001790 33449587

[4] Swampillai AJ, Sherman T, Garg A, Tan IJ, Lim KS. Secondary pigmentary glaucoma following cosmetic laser treatment to alter iris colour. Cont Lens Anterior Eye. 2023 Apr;46(2):101754. 10.1016/j.clae.2022.101754 36175318

[5] Flores-Márquez A, Moreno-Gutiérrez J, Chinchurreta-Capote A, García-Martín F, Rocha-de-Lossada C. Laser-induced maculopathy after iris depigmentation cosmetic treatment. Can J Ophthalmol. 2023 Feb;58(1):e29–e31. 10.1016/j.jcjo.2022.05.012 35809624

[6] Alió JL, Rodriguez AE, Toffaha BT, Piñero DP, Moreno LJ (2011). Femtosecond-assisted keratopigmentation for functional and cosmetic restoration in essential iris atrophy. J Cataract Refract Surg.

[7] Ferrari F, Morin L (2015). [Description of a new method of changing eye color: case report of aesthetic annular keratopigmentation (AAK)]. J Fr Ophtalmol.

[8] Ziegler SL (1922). Multicolor Tattooing of the cornea. Trans Am Ophthalmol Soc.

[9] Alio JL, Rodriguez AE, Toffaha BT (2011). Keratopigmentation (corneal tattooing) for the management of visual disabilities of the eye related to iris defects. Br J Ophthalmol.

[10] Hirsbein D, Gardea E, Brasseur G, Muraine M (2008). [Corneal tattooing for iris defects]. J Fr Ophtalmol.

[11] Roy JN (1938). TATTOOING OF THE CORNEA. Can Med Assoc J.

[12] Pitz S, Jahn R, Frisch L, Duis A, Pfeiffer N (2002). Corneal tattooing: an alternative treatment for disfiguring corneal scars. Br J Ophthalmol.

[13] Kymionis GD, Ide T, Galor A, Yoo SH (2009). Femtosecond-assisted anterior lamellar corneal staining-tattooing in a blind eye with leukocoria. Cornea.

[14] Laria C, Alió JL, Piñero DN (2010). Intrastromal corneal tattooing as treatment in a case of intractable strabismic diplopia (double binocular vision). Binocul Vis Strabismus Q.

[15] Alio JL, Rodriguez AE, Toffaha BT, El Aswad A (2012). Femtosecond-assisted keratopigmentation double tunnel technique in the management of a case of Urrets-Zavalia syndrome. Cornea.

[16] D’Oria F, Alio JL, Rodriguez AE, Amesty MA, Abu-Mustafa SK (2021). Cosmetic Keratopigmentation in sighted eyes: medium- and long-term clinical evaluation. Cornea.

[17] Ferrari F, van Haselen R (2018). The Safety and Effectiveness of a Novel Annular Keratopigmentation Method: a Case Report. Case Rep Ophthalmol.

[18] Burk SE (2001). Prosthetic iris implantation for congenital, traumatic, or functional iris deficiencies. J Cataract Refract Surg.

[19] Yildirim Y (2016). Evaluation of Color-Changing effect and complications after nd: YAG Laser Application on Iris Surface. Med Sci Monit.

[20] Anderson JE, Grippo TM, Sbeity Z, Ritch R (2010). Serious complications of cosmetic NewColorIris implantation. Acta Ophthalmol.

[21] Ali MH, Traish AS (2016). Elevated intraocular pressure and endothelial cell loss following Iris Color Change. JAMA Ophthalmol.

[22] Arthur SN, Wright MM, Kramarevsky N, Kaufman SC, Grajewski AL (2009). Uveitis-glaucoma-hyphema syndrome and corneal decompensation in association with cosmetic iris implants. Am J Ophthalmol.

[23] Amesty MA, Rodriguez AE, Hernández E, De Miguel MP, Aliò JL (2016). Tolerance of Micronized Mineral Pigments for Intrastromal Keratopigmentation: a histopathology and immunopathology experimental study. Cornea.

[24] Hasani H, Es’haghi A, Rafatnia S, Alilou S, Abolmaali M (2020). Keratopigmentation: a comprehensive review. Eye.

